# Supplementary material for: Trends in cognitive outcomes in middle-aged Americans across three birth cohorts
Source: PLoS One. 2025 Dec 5;20(12):e0338368. doi: 10.1371/journal.pone.0338368 (PMC12680256; doi:10.1371/journal.pone.0338368)
Supplement: S1 Table — Note: HRS = Health and Retirement Study. Adapted from Langa, K. M. (2020). Langa-Weir classification of cognitive function (1995 Onward). Survey Research Center Institute for Social Research, University of Michigan, p. 5. (DOCX) [file pone.0338368.s001.docx]

**Supplementary Table 1**

*Cut-points on the HRS Cognitive Score Self-Respondent 27-point Scale and Proxy-Respondent Scale*

|  | Self-Respondent | Proxy Respondent | |
| --- | --- | --- | --- |
| Cognitive Function | 27-point scale  (1995-) | 9-point scale  (1995-1998) | 11-point scale (2000-) |
| Normal | 12-27 | 0-2 | 0-2 |
| CIND | 7-11 | 3-4 | 3-5 |
| Dementia | 0-6 | 5-9 | 6-11 |

*Note:* HRS = Health and Retirement Study. Adapted from Langa, K. M. (2020). Langa-Weir classification of cognitive function (1995 Onward). *Survey Research Center Institute for Social Research, University of Michigan*, p. 5.
