# Supplementary material for: Trends in cognitive outcomes in middle-aged Americans across three birth cohorts
Source: PLoS One. 2025 Dec 5;20(12):e0338368. doi: 10.1371/journal.pone.0338368 (PMC12680256; doi:10.1371/journal.pone.0338368)
Supplement: S2 Table — Note. Analyses incorporate survey weights, strata, and clusters to account for the complex HRS survey design. (DOCX) [file pone.0338368.s002.docx]

**Supplementary Table 2**

*Proportion of Self Versus Proxy Respondents by Birth Cohort over Follow-up*

|  | Participants, weighted row % | | | | | | | | *p* |
| --- | --- | --- | --- | --- | --- | --- | --- | --- | --- |
|  | Overall  (N=7,852) | | War Babies,  born 1942-1947  (N=1,926) | | Early Baby Boomers, born 1948-1953  (N=2,480) | | Mid Baby Boomers,  born 1954-1959  (N=3,446) | |  |
| Visit type | Self | Proxy | Self | Proxy | Self | Proxy | Self | Proxy |  |
| Baseline | 95.5 | 4.5 | 94.3 | 5.7 | 93.9 | 6.1 | 98.2 | 1.8 | <.001 |
| Visit 2 | 96.1 | 3.9 | 94.0 | 6.0 | 95.8 | 4.2 | 98.3 | 1.7 | <.001 |
| Visit 3 | 95.7 | 4.3 | 92.6 | 7.4 | 96.7 | 3.3 | 97.8 | 2.2 | <.001 |
| Visit 4 | 96.0 | 4.0 | 94.3 | 5.7 | 95.8 | 4.2 | 97.9 | 2.1 | <.001 |
| Visit 5 | 97.0 | 3.0 | 96.1 | 3.9 | 96.7 | 3.3 | 98.2 | 1.8 | <.001 |

*Note.* Analyses incorporate survey weights, strata, and clusters to account for the complex HRS survey design.
