# Supplementary material for: Trends in cognitive outcomes in middle-aged Americans across three birth cohorts
Source: PLoS One. 2025 Dec 5;20(12):e0338368. doi: 10.1371/journal.pone.0338368 (PMC12680256; doi:10.1371/journal.pone.0338368)
Supplement: S3 Table — Note. GED = General Educational Development test; SD = standard deviation. (DOCX) [file pone.0338368.s003.docx]

**Supplementary Table 3**

*Distribution of Participant Characteristics by Group-Based Trajectory*

|  | Group-based trajectory  No. (column %) | | | |  |
| --- | --- | --- | --- | --- | --- |
| Characteristic, No. (weighted %) | 1  (n=815) | 2  (n=2,931) | 3  (n=3,325) | 4  (n=781) | *p* |
| Birth cohort |  |  |  |  | .004 |
| War Babies | 162 (34.5) | 653 (32.7) | 849 (32.6) | 262 (39.8) |  |
| Early Baby Boomers | 238 (32.0) | 887 (31.1) | 1079 (31.2) | 276 (32.4) |  |
| Mid Baby Boomers | 413 (33.5) | 1390 (36.3) | 1397 (36.2) | 243 (27.9) |  |
| Age, mean year (SD) | 53.2 (1.6) | 53.2 (1.5) | 53.0 (1.4) | 52.8 (1.3) | <.001 |
| Gender |  |  |  |  |  |
| Woman | 45.3 | 43.3 | 46.6 | 57.1 | <.001 |
| Man | 54.7 | 56.7 | 53.4 | 42.9 |  |
| Race/ethnicity |  |  |  |  | <.001 |
| White non-Latino | 52.3 | 69.9 | 84.9 | 93.8 |  |
| Black non-Latino | 25.0 | 13.6 | 6.4 | 2.4 |  |
| Other non-Latino | 6.3 | 5.1 | 3.6 | 1.3 |  |
| Latino | 16.4 | 11.3 | 5 | 2.4 |  |
| Educational attainment |  |  |  |  | <.001 |
| Less than high school | 43.2 | 18.2 | 6.8 | 2.3 |  |
| High school or GED | 29.4 | 32.1 | 23.6 | 12.2 |  |
| Some college | 21.2 | 31.1 | 29.4 | 25.6 |  |
| College or higher | 6.1 | 18.7 | 40.2 | 59.9 |  |
| Income, quartile, $ |  |  |  |  | <.001 |
| ≤$10,559 | 36.1 | 18 | 8.8 | 4.2 |  |
| >$10,559, ≤$22,916 | 28.9 | 23.1 | 16 | 10.7 |  |
| >$22,916, ≤$44,083 | 22.4 | 32.7 | 30 | 26.4 |  |
| >44,083 | 12.6 | 26.3 | 45.2 | 58.6 |  |
| Net worth, quartile, $ |  |  |  |  | <.001 |
| ≤$17,527 | 49.8 | 30.4 | 18.8 | 10.9 |  |
| >$17,527, ≤$71,657 | 30.1 | 28 | 24 | 17.8 |  |
| >$71,657, ≤$188,325 | 11.5 | 22.3 | 27.3 | 29.4 |  |
| >188,325 | 8.7 | 19.2 | 30 | 41.9 |  |
| Chronic medical conditions |  |  |  |  |  |
| Hypertension | 43.7 | 35.5 | 30.8 | 20.7 | <.001 |
| Stroke | 3.1 | 2.6 | 1.5 | 1.1 | .0041 |
| Diabetes | 15.2 | 12.6 | 8.5 | 6.1 | <.001 |
| Cardiac disease | 11.8 | 9.9 | 8.1 | 4.2 | <.001 |
| Body mass index |  |  |  |  |  |
| <18.5 | 2.0 | 1.3 | 1.1 | 1.0 | .0015 |
| 18.5-24.9 | 25.2 | 25.6 | 26.5 | 34.5 |  |
| 25-29.9 | 35.4 | 39.1 | 41.4 | 40.7 |  |
| ≥30 | 37.4 | 34 | 31 | 23.7 |  |

*Note.* GED = General Educational Development test; SD = standard deviation.
