# Supplementary material for: Trends in cognitive outcomes in middle-aged Americans across three birth cohorts
Source: PLoS One. 2025 Dec 5;20(12):e0338368. doi: 10.1371/journal.pone.0338368 (PMC12680256; doi:10.1371/journal.pone.0338368)
Supplement: S4 Table — Note. CI = confidence interval. Relative risk ratios calculated using multi-nominal logistic regression models. Statistically significant risk ratios are in bold. (DOCX) [file pone.0338368.s004.docx]

**Supplementary Table 4**

*Association of Birth Cohort with Group-Based Trajectory, Unadjusted*

|  | **Trajectory groups (reference = Group 4)** | | |
| --- | --- | --- | --- |
|  | Group 1  relative risk ratio (95% CI) | Group 2  relative risk ratio (95% CI) | Group 3  relative risk ratio (95% CI) |
| Birth cohort |  |  |  |
| War Babies | Reference | Reference | Reference |
| Early Baby Boomers | 1.14 (0.83-1.56) | 1.17 (0.92-1.49) | 1.18 (0.96-1.44) |
| Mid Baby Boomers | 1.38 (0.95-2.02) | **1.58 (1.19-2.11)** | **1.58 (1.28-1.95)** |

*Note.* CI = confidence interval. Relative risk ratios calculated using multi-nominal logistic regression models. Statistically significant risk ratios are in bold.
