# Supplementary material for: Trends in cognitive outcomes in middle-aged Americans across three birth cohorts
Source: PLoS One. 2025 Dec 5;20(12):e0338368. doi: 10.1371/journal.pone.0338368 (PMC12680256; doi:10.1371/journal.pone.0338368)
Supplement: S5 Table — Note. CI = confidence interval; GED = General Educational Development test. Relative risk ratios calculated using multi-nominal logistic regression models. Statistically significant risk ratios are in bold. (DOCX) [file pone.0338368.s005.docx]

**Supplementary Table 5**

*Association of Birth Cohort and Other Participant Characteristics with Group-Based Trajectory, Adjusted*

|  | **Trajectory groups (reference = Group 4)** | | |
| --- | --- | --- | --- |
|  | Group 1  relative risk ratio (95% CI) | Group 2  relative risk ratio (95% CI) | Group 3  relative risk ratio (95% CI) |
| Birth cohort |  |  |  |
| War Babies | Reference | Reference | Reference |
| Early Baby Boomers | **1.47 (1.08-2.00)** | **1.35 (1.08-1.69)** | **1.28 (1.05-1.56)** |
| Mid Baby Boomers | 1.29 (0.91-1.83) | **1.55 (1.18-2.04)** | **1.60 (1.31-1.97)** |
| Age, years | **1.16 (1.08-1.24)** | 1.12 (1.06-1.18) | 1.04 (0.99-1.09) |
| Gender |  |  |  |
| Man | **2.11 (1.48-3.01)** | **2.09 (1.67-2.62)** | **1.61 (1.35-1.92)** |
| Woman | Reference | Reference | Reference |
| Race/ethnicity |  |  |  |
| White non-Latino | Reference | Reference | Reference |
| Black non-Latino | **9.92 (6.08-16.18)** | **5.34 (3.61-7.88)** | **2.43 (1.69-3.49)** |
| Other | **8.34 (3.45-20.18)** | **5.02 (2.45-10.29)** | **2.85 (1.49-5.43)** |
| Latino | **3.91 (2.39-6.41)** | **3.33 (2.06-5.36)** | **1.71 (1.1-2.67)** |
| Educational attainment |  |  |  |
| Less than high school | **80.98 (47.63-137.67)** | **15.53 (9.65-25.01)** | **3.5 (2.12-5.8)** |
| High school or GED | **19.86 (13.12-30.06)** | **8.19 (6.1-11.02)** | **2.86 (2.19-3.72)** |
| Some college | **6.05 (4.18-8.75)** | **3.48 (2.79-4.35)** | **1.61 (1.27-2.03)** |
| College or higher | Reference | Reference | Reference |
| Net worth, quartile, $ |  |  |  |
| ≤$17,527 | **4.94 (2.82-8.65)** | **2.35 (1.61-3.42)** | **1.54 (1.09-2.18)** |
| >$17,527, ≤$71,657 | **2.90 (1.80-4.66)** | **1.73 (1.31-2.29)** | **1.38 (1.06-1.78)** |
| >$71,657, ≤$188,325 | **1.26 (0.70-2.26)** | **1.28 (1.00-1.63)** | **1.17 (0.95-1.45)** |
| >188,325 | Reference | Reference | Reference |
| Chronic medical conditions |  |  |  |
| Hypertension | **1.85 (1.40-2.45)** | **1.41 (1.15-1.73)** | **1.32 (1.08-1.62)** |
| Stroke | 1.18 (0.39-3.54) | 1.39 (0.55-3.52) | 0.97 (0.36-2.59) |
| Diabetes | 1.06 (0.64-1.77) | 1.13 (0.74-1.71) | 0.92 (0.62-1.37) |
| Cardiac disease | 1.63 (0.88-3.03) | 1.58 (0.93-2.67) | 1.51 (0.85-2.69) |
| Body mass index |  |  |  |
| <18.5 | 0.85 (0.31-2.31) | 0.86 (0.39-1.92) | 1.10 (0.47-2.61) |
| 18.5-24.9 | Reference | Reference | Reference |
| 25-29.9 | 0.70 (0.48-1.02) | 0.84 (0.66-1.07) | 1.02 (0.82-1.27) |
| ≥30 | 0.83 (0.60-1.15) | 0.96 (0.75-1.24) | 1.15 (0.90-1.45) |

*Note.* CI = confidence interval; GED = General Educational Development test. Relative risk ratios calculated using multi-nominal logistic regression models. Statistically significant risk ratios are in bold.
