# Supplementary material for: Trends in cognitive outcomes in middle-aged Americans across three birth cohorts
Source: PLoS One. 2025 Dec 5;20(12):e0338368. doi: 10.1371/journal.pone.0338368 (PMC12680256; doi:10.1371/journal.pone.0338368)
Supplement: S6 Table — Note. CI = confidence interval; HRS = Health and Retirement Study. HRS cognitive score change calculated using mixed-effects models. Statistically significant differences in HRS scores are in bold. (DOCX) [file pone.0338368.s006.docx]

**Supplementary Table 6**

*Association Between Baseline Characteristics and Intercept and Slope of HRS Cognitive Score Change, Unadjusted*

|  | Intercept, HRS Score Change  (95% CI) | Slope, HRS Score Change  (95% CI) |
| --- | --- | --- |
| Birth Cohort |  |  |
| War Babies | Reference | Reference |
| Early Baby Boomers | **-0.305 (-0.484, -0.126)** | 0.008 (-0.021, 0.036) |
| Mid Baby Boomers | **-1.014 (-1.187, -0.840)** | **0.112 (0.083, 0.141)** |

*Note.* CI = confidence interval; HRS = Health and Retirement Study. HRS cognitive score change calculated using mixed-effects models. Statistically significant differences in HRS scores are in bold.
