# Supplementary material for: Trends in cognitive outcomes in middle-aged Americans across three birth cohorts
Source: PLoS One. 2025 Dec 5;20(12):e0338368. doi: 10.1371/journal.pone.0338368 (PMC12680256; doi:10.1371/journal.pone.0338368)
Supplement: S7 Table — Note. CIND = Cognitive impairment no dementia. CI = confidence interval. Statistically significant hazard ratios are in bold. (DOCX) [file pone.0338368.s007.docx]

**Supplementary Table 7**

*Association of Birth Cohort with Persistent CIND, Unadjusted*

|  | Persistent CIND,  0-5 years  Hazard Ratio (95% CI) | Persistent CIND,  >5 years  Hazard Ratio (95% CI) |
| --- | --- | --- |
| Cohort |  |  |
| War Babies | Reference | Reference |
| Early Baby Boomers | 0.94 (0.66, 1.35) | **0.50 (0.27, 0.92)** |
| Mid Baby Boomers | 0.84 (0.59, 1.19) | **0.37 (0.19, 0.73)** |

*Note.* CIND = Cognitive impairment no dementia. CI = confidence interval. Statistically significant hazard ratios are in bold.
