# Supplementary material for: Trends in cognitive outcomes in middle-aged Americans across three birth cohorts
Source: PLoS One. 2025 Dec 5;20(12):e0338368. doi: 10.1371/journal.pone.0338368 (PMC12680256; doi:10.1371/journal.pone.0338368)
Supplement: S1 Fig — Note. CIND = Cognitive impairment no dementia. HRS = Health and Retirement Study. The figure shows the incidence of persistent CIND across the three birth cohorts, stratified by race/ethnicity. Cumulative incidences were determined using Kaplan-Meier curves. Analyses incorporated survey weights, strata, and clusters to account for the complex HRS survey design. (DOCX) [file pone.0338368.s008.docx]

**Supplementary Figure 1**

*Kaplan Meier Curves for Persistent CIND by Birth Cohort, Stratified by Race/Ethnicity*

|  |  |
| --- | --- |
| White non-Latino | Black non-Latino |
|  |  |
| Other non-Latino | Latino |
