# Supplementary material for: Trends in cognitive outcomes in middle-aged Americans across three birth cohorts
Source: PLoS One. 2025 Dec 5;20(12):e0338368. doi: 10.1371/journal.pone.0338368 (PMC12680256; doi:10.1371/journal.pone.0338368)
Supplement: Attachment — Submitted filename: ADRD MS Resubmission Response Letter FINAL.docx [file pone.0338368.s009.docx]

Rebecca T. Brown, MD, MPH

Associate Professor of Medicine

3615 Chestnut Street

Philadelphia, PA 19104

Tel: (215) 573-2021

Rebecca.Brown@pennmedicine.upenn.edu

July 3, 2025

Amruta Deshpande, Academic Editor
*PLOS One*

Dear Dr. Deshpande,

We thank the Editor and Reviewers for their comments and appreciate the opportunity to address the comments and revise our manuscript. Below, please find item-by-item responses to the comments, which are included verbatim. All page, paragraph, and line numbers refer to locations in the revised manuscript and online-only supplement. We have uploaded a clean copy of the revised manuscript as well as a copy of the manuscript with tracked changes.

**Journal requirements:**

1. When submitting your revision, we need you to address these additional requirements.
   a. Please ensure that your manuscript meets PLOS ONE's style requirements, including those for file naming. The PLOSONE style templates can be found at <https://journals.plos.org/plosone/s/file?id=wjVg/PLOSOne_formatting_sample_main_body.pdf> and

[https://journals.plos.org/plosone/s/file?id=ba62/PLOSOne_formatting_sample_title_authors_affiliations.pdf](https://urldefense.com/v3/__https:/track.editorialmanager.com/CL0/https:*2F*2Fjournals.plos.org*2Fplosone*2Fs*2Ffile*3Fid=ba62*2FPLOSOne_formatting_sample_title_authors_affiliations.pdf/1/010f0196f3705e4b-e42a7692-db3c-4026-800e-f8e2fee3e897-000000/n9_bLEZ7s6zD0dJzYftqKOH_q3ZMqkHSP4x0F-mxv9c=212__;JSUlJSUlJQ!!IBzWLUs!TsrgNTqUv_34eklYepbLp-t4d1xwdHGKOLa1fjZ_QOPg45ICfXgPIiU8LjCCmoBCw3hCGi_XcrqBG5QCRg_6KLQpDhWj8xWO$)

b. **Response:** We have revised the manuscript to meet PLOS ONE’s style requirements.

2. Thank you for stating in your Funding Statement: [This work was supported by the National Institute on Aging at the National Institutes of Health (grant number R01AG070885 to R.T.B.; grant numbers K23AG065442 and K23AG065442-03S1 to S.D.S.; P30AG072979 to C.T.M) and Penn Institute on Aging (no grant number to C.T.M.). The Health and Retirement Study is supported by the National Institute on Aging (grant number U01AG009740) and performed at the Institute for Social Research, University of Michigan.].

Please provide an amended statement that declares *all* the funding or sources of support (whether external or internal to your organization) received during this study, as detailed online in our guide for authors at [http://journals.plos.org/plosone/s/submit-now](https://urldefense.com/v3/__http:/journals.plos.org/plosone/s/submit-now__;!!IBzWLUs!TsrgNTqUv_34eklYepbLp-t4d1xwdHGKOLa1fjZ_QOPg45ICfXgPIiU8LjCCmoBCw3hCGi_XcrqBG5QCRg_6KLQpDki3KOOh$).  Please also include the statement “There was no additional external funding received for this study.” in your updated Funding Statement.

Please include your amended Funding Statement within your cover letter. We will change the online submission form on your behalf.

a. **Response:** We have revised the funding statement as requested and have included the amended Funding Statement within our cover letter.

**Responses to Additional Editor Comments:**

1. Mayor revisión. Work on comments given by reviewers and submit the revised manuscript.
   a. **Response:** We appreciate the opportunity to respond to the reviewer comments below.

**Responses to Reviewer 1:**

1. Dear Authors
   Your article sheds light to a valid concept and work is well done. I believe you can consider these extra changes to make your excellent work perfect:
   a. **Response:** We thank the reviewer for these comments. We have responded to the specific questions and comments below.
2. Proxy Respondent Calibration: Consider a sensitivity analysis excluding proxy respondents to assess the robustness of cohort comparisons, given changes in HRS data collection procedures after 2006.
   a. **Response:** We agree that this is an important consideration and have now completed sensitivity analyses for the linear mixed-effects and Cox regression models that exclude proxy respondents, as follows. For each participant-wave (i.e., longitudinal repeated measures), we excluded those waves in which a proxy responded on behalf of the participant. In total, 211 individuals for whom proxies completed all survey waves were excluded, for a final sample of 7,641. In adjusted mixed effects models after proxy waves were excluded, the baseline cognitive scores for both Early Baby Boomers and Mid Baby Boomers relative to War Babies were slightly higher when proxies were excluded than when proxies were included, with respective values of -0.310 (95% CI, -0.467, -0.152) versus -0.316 (95% CI, -0.480, -0.151), and -0.658 (95% CI, -0.816, -0.500) versus -0.691 (95% CI, -0.854, -0.527).

Similarly, the rate of cognitive decline (slope) for Early Baby Boomers compared to War Babies was slower when proxies were excluded compared to when they were included, with a value of 0.062 (95% CI, 0.022, 0.102) without proxies versus 0.004 (95% CI, -0.025, 0.033) with proxies. In other words, after excluding the proxies, the difference in slope between Early Baby Boomers and War Babies became more positive. A similar pattern was observed for Mid Baby Boomers compared to War Babies, with a value of 0.187 without proxies (95% CI, 0.139, 0.235) versus 0.122 with proxies (95% CI, 0.092, 0.152).

Thus, while there is a relatively small absolute change in the value of the intercepts and slopes, the direction of the findings is consistent with those of the primary analyses. These findings suggest that the cohort comparisons are robust despite changes in HRS data collection procedures after 2006. As expected, the findings show that when individuals with proxy respondents are excluded, baseline cognitive scores are higher and cognitive decline is slower, consistent with the excluded individuals having lower cognitive scores at baseline and over follow-up.

In the unadjusted and adjusted Cox regression models excluding proxies, some previously significant findings were no longer significant (see eTable 7 and Table 2 for estimates with and without proxies). This is expected, as proxies were often interviewed due to respondents’ impaired cognitive function. Excluding these cases reduced the number of events (i.e., episodes of persistent CIND) from 388 to 332.

*Association of Birth Cohort with Persistent CIND, with and without Proxies*

|  |  | Persistent CIND,  0-5 years  Hazard Ratio (95% CI) | Persistent CIND,  >5 years  Hazard Ratio (95% CI) |
| --- | --- | --- | --- |
|  | Cohort, overall | Unadjusted | Unadjusted |
| Including proxies | War Babies | Reference | Reference |
|  | Early Baby Boomers | 0.94 (0.66, 1.35) | **0.50 (0.27, 0.92)** |
|  | Mid Baby Boomers | 0.84 (0.59, 1.19) | **0.37 (0.19, 0.73)** |
|  |  |  |  |
| Excluding proxies | War Babies | Reference | Reference |
|  | Early Baby Boomers | 1.05 (0.73, 1.53) | 0.53 (0.23, 1.24) |
|  | Mid Baby Boomers | 0.86 (0.59, 1.26) | 0.56 (0.25, 1.26) |

*Note.* CIND = Cognitive impairment no dementia. CI = confidence interval. Statistically significant hazard ratios are in bold.

*Association between Birth Cohort and Risk of Persistent CIND, Stratified by Race/Ethnicity, with and without Proxies*

|  |  | Persistent CIND,  0-5 years  Hazard Ratio (95% CI) | Persistent CIND,  >5 years  Hazard Ratio (95% CI) |
| --- | --- | --- | --- |
| Including proxies | Birth Cohort, Stratified by Race/Ethnicity | Adjusted | Adjusted |
|  | White non-Latino |  |  |
|  | War Babies | Reference | Reference |
|  | Early Baby Boomers | **4.38 (2.62, 7.33)** | **0.08 (0.02, 0.23)** |
|  | Mid Baby Boomers | **2.68 (1.67, 4.29)** | **0.05 (0.01, 0.21)** |
|  | Black non-Latino |  |  |
|  | War Babies | Reference | Reference |
|  | Early Baby Boomers | 1.02 (0.49, 2.12) | 4.50 (0.56, 36.00) |
|  | Mid Baby Boomers | 1.08 (0.58, 2.02) | 1.83 (0.23, 14.33) |
|  | Other non-Latino |  |  |
|  | War Babies | Reference | Reference |
|  | Early Baby Boomers | 1.17 (0.25, 5.41) | Not estimable |
|  | Mid Baby Boomers | 1.09 (0.27, 4.50) | Not estimable |
|  | Latino |  |  |
|  | War Babies | Reference | Reference |
|  | Early Baby Boomers | 1.01 (0.55, 1.87) | 0.20 (0.04, 1.09) |
|  | Mid Baby Boomers | **0.45 (0.20, 1.00)** | 0.40 (0.11, 1.40) |
|  |  |  |  |
| Excluding proxies | Birth Cohort, Stratified by Race/Ethnicity | Adjusted | Adjusted |
|  | White non-Latino |  |  |
|  | War Babies | Reference | Reference |
|  | Early Baby Boomers | 1.28 (0.71, 2.32) | **0.03 (0.00, 0.21)** |
|  | Mid Baby Boomers | 0.65 (0.35, 1.21) | **0.29 (0.08, 1.10)** |
|  | Black non-Latino |  |  |
|  | War Babies | Reference | Reference |
|  | Early Baby Boomers | 1.09 (0.51, 2.34) | 2.20 (0.40, 12.19) |
|  | Mid Baby Boomers | 1.59 (0.84, 3.01) | 1.12 (0.23, 5.47) |
|  | Other non-Latino |  |  |
|  | War Babies | Reference | Reference |
|  | Early Baby Boomers | 1.32 (0.32, 5.48) | Not estimable |
|  | Mid Baby Boomers | 1.47 (0.45, 4.76) | Not estimable |
|  | Latino |  |  |
|  | War Babies | Reference | Reference |
|  | Early Baby Boomers | 0.98 (0.51, 1.90) | Not estimable |
|  | Mid Baby Boomers | **0.41 (0.18, 0.92)** | Not estimable |

We have edited the manuscript to include these findings, as follows:

**Methods, page 13, paragraph 1: “**In sensitivity analyses for both the linear mixed-effects and Cox regression models, we excluded proxy respondents to assess the robustness of the cohort comparisons, given changes in HRS data collection procedures over time.”

**Results, page 18, paragraph 2:** “Sensitivity analyses excluding proxies showed a similar pattern of results; baseline cognitive scores were slightly higher and slopes of decline were lower compared to the primary analyses, consistent with the more prevalent use of proxies among respondents with cognitive impairment. The number of CIND cases decreased from 388 to 332 after excluding proxies, and several unadjusted and adjusted hazard ratios were no longer statistically significant (analyses not shown).”

3. CIND Onset Estimation: Clarify or address the potential misclassification of CIND onset due to the assumption of linear cognitive decline between biennial assessments. A subgroup analysis excluding participants with stroke could help.
   a. **Response:** We agree that this is an important consideration and have examined the number of strokes that occurred during the same interval as incident persistent CIND. Persistent CIND was defined as having two consecutive interviews indicating impaired cognitive function, with the time of onset estimated between the interview showing impairment and the prior interview in which cognitive function was normal. If a stroke occurred during this interval, it could violate the assumption of linear cognitive change. Among the 388 persistent CIND cases in our study, only 27 had a stroke in the same interval. Therefore, the potential impact of stroke on the linearity assumption is minimal. We now address this in the main text as follows:

**Discussion, page 27, paragraph 1:** “Because interviews are biennial, we were unable to observe the exact date of CIND onset. Thus, we assumed a linear cognitive function change between the dates of interview before and after CIND and interpolated the date of CIND onset. Any non-linear slope of cognitive decline that occurred between two interviews, such as a sudden drop in cognitive score (e.g., after a health event such as a stroke or a traumatic brain injury) would violate this assumption. Thus, the interpolated date of CIND onset for individuals with a sudden drop in cognitive score may not be accurately specified between the two interviews. However, among the 388 persistent CIND cases, only 27 had a stroke in the same interval, suggesting limited impact on our findings.”

4. Supplementary Material Integration: Summarize key findings from supplementary tables (e.g., adjusted trajectory group membership) in the main text for better accessibility.
   a. **Response:** We now summarize key findings from the supplementary tables in the main text, as follows:

**Results, page 16, paragraph 1:** “The birth cohorts made up significantly different proportions of each trajectory group (Supplementary Table 3). War Babies comprised the largest proportion of Group 1 (34.5%) and Group 4 (39.8%), the groups with the lowest baseline cognitive score and the highest baseline cognitive score, respectively. Mid Boomers made up the largest proportion of the two middle groups (Group 2, 36.3% and Group 3, 36.2%) and the smallest proportion of the group with the highest baseline cognitive score (Group 4, 27.9%). Early Boomers comprised similar proportions of each trajectory group, ranging from 31.1% to 32.4%.”

**Results, page 17, paragraph 1**: “Consistent with these descriptive findings, in unadjusted multinomial logistic regression analyses in which the group with the highest cognitive baseline was the reference, Mid Boomers had a significantly higher relative risk of being in the two middle trajectory groups compared to War Babies (relative risk [RR] for Group 2 membership, 1.58, 95% CI, 1.19-2.11; RR for Group 3 membership, 1.58, 95% CI, 1.28-1.95; Supplementary Table 4). Adjusted analyses were also consistent with descriptive findings: Early Boomers had a significantly higher risk of being in the three groups with the lowest cognitive baselines compared to War Babies (RR for Group 1, 1.47 [95% CI, 1.08-2.00]; RR for Group 2, 1.35 [95% CI, 1.08-1.69]; RR for Group 3, 1.28 [95% CI, 1.05-1.56]), while Mid Boomers had a significantly higher risk of being in the two middle groups (RR for Group 2, 1.55 [95% CI, 1.18-2.04]; RR for Group 3, 1.60 [95% CI, 1.31-1.97]; Supplementary Table 5).”

**Results, page 17, paragraph 2**: “Among demographic characteristics in the adjusted multinomial logistic regression analyses, older (versus younger) individuals had a significantly higher risk of being in the group with the lowest cognitive baseline versus that with the highest cognitive baseline (Supplementary Table 5).”

5. Sample Size Caveats: Explicitly acknowledge the limited statistical power in racial/ethnic minority subgroup analyses when interpreting null results.
   a. **Response:** We now acknowledge the limited statistical power in subgroup analyses among racial/ethnic minorities in our interpretation of the null results, as follows:

**Results, page 22, paragraph 1:** “Of note, the null results for subgroup analyses among respondents identifying as Black non-Latino, Other non-Latino, and Latino may reflect limited statistical power.”

**Responses to Reviewer 2:**

1. This manuscript addresses an important topic by examining cognitive outcomes using data from the Health and Retirement Study (HRS). While the use of a large, nationally representative dataset and the authors’ effort to consider weights and competing risks, such as mortality, are commendable, I have significant concerns regarding the validity of the core analytic approach and the lack of clarity in model specification. My comments below outline specific concerns that would require significant clarification and methodological refinement for the study to meet the standards of rigor and interpretability expected for publication.
   a. **Response:** We thank the reviewer for these comments and have responded to the specific comments below.
2. The introduction would benefit from revision to provide a clearer and more compelling rationale for the study. Specifically, it is not well explained why differences in cognitive outcomes across individual birth cohorts should be expected (and in which direction). A stronger theoretical or empirical justification for examining cohort effects is needed to frame the research question. Additionally, the authors refer to examining “cognitive trajectories and incidence of cognitive decline in the form of “cognitive impairment no dementia” (CIND, often a precursor of dementia),” but no incidence rates appear to be reported in the manuscript. If incidence was not estimated, this terminology should be removed or revised to more accurately reflect the outcomes analyzed (e.g. the relative hazard of CIND).
   a. **Response:** We agree that the introduction would be strengthened by these additions and have expanded it to more clearly justify the examination of cohort effects. In addition, we have clarified the language around incidence/risk of CIND in the introduction and have added incidence rates in the results section, as follows:

**Introduction, page 4, paragraph 1:** “At the same time, there is growing evidence that health status has worsened in middle-aged Americans since the 1990s compared to earlier birth cohorts, including increases in the prevalence of chronic medical conditions that are risk factors for AD/ADRD (15-18). This trend suggests the need to look beyond individual-level risk factors to understand how one’s birth cohort may influence risk for health outcomes, possibly through exposures in the broader social or physical environment. This would allow for population-focused efforts to improve health outcomes for this age group and potentially reduce overall dementia burden. Yet little is known about whether cognitive outcomes have worsened in middle-aged people over this time period, as have rates of chronic conditions.

While the causes of the recent declines in health status in middle-aged Americans are still unclear, several studies have shown that middle-aged adults with lower socioeconomic status (SES), including lower education, income, and net worth, are at highest risk for these health declines(15-18). Moreover, SES disparities in prevalence of chronic conditions and mortality risk in the U.S. have worsened substantially over the past decades, a trend occurring in parallel with economic stagnation for lower-income households (19, 20). Based on these trends, researchers hypothesize that lower SES is leading to worsening health status in middle-aged adults in more recent birth cohorts, possibly through exposures in the social and physical environment (21-23). A growing number of studies have linked such environmental factors to risk of cognitive decline and AD/ADRD in older adults,(24-30) and these factors could also be impacting cognitive trajectories and risk of AD/ADRD in middle age. However, despite increases in the prevalence of AD/ADRD risk factors in more recent birth cohorts compared to earlier birth cohorts, little is known about trends in cognitive outcomes in middle-aged adults.

Several prior studies have examined trends in outcomes related to cognition; one study showed that from 1999 to 2017, mortality from neurodegenerative diseases increased 88% in people ages 55-64 years (31). Several cross-sectional studies have also shown that the prevalence of difficulty performing cognitively complex instrumental activities of daily living (IADLs) remained stable or increased in middle-aged people over the past 2-3 decades (17, 18, 32). Another study found that the prevalence of cognitive limitations among individuals ages 55-69 was stable from 1998 to 2014, but showed substantial disparities by socioeconomic status (33). Taken together, these studies suggest that the prevalence of cognitive impairment in middle age has remained stable or worsened over the past two decades, particularly among those with lower socioeconomic status. However, these studies assessed prevalent cognitive impairment. Thus, they do not distinguish between longstanding impairments due to congenital conditions, trauma, or early health events versus cognitive changes that develop in middle age and may have different clinical implications.

To examine if cognitive outcomes have worsened in more recent birth cohorts of middle-aged adults compared to those born earlier, we examined cognitive trajectories and incidence and hazards of cognitive decline in the form of “cognitive impairment no dementia” (CIND, often a precursor of dementia) in middle age among three birth cohorts: War Babies (born 1942-1947); Early Baby Boomers (born 1948-1953); and Mid Baby Boomers (born 1954-1959). We also examined how cognitive trajectories and hazards of CIND differ among subgroups including persons with lower socioeconomic status and racial/ethnic minorities.”

**Methods, page X, paragraph Y:** “To compare the incidence and hazards of persistent CIND across the three birth cohorts, we used Kaplan-Meier curves. We accounted for the competing risk of death in two ways. First, we censored at the time of death to model cause-specific hazard in Cox regressions; second, we used Fine and Gray’s subdistribution hazard model (35). For the Cox regression models, we checked the proportional hazard assumption. For variables violating the assumption, we divided the total length of follow-up into two periods and estimated separate hazard ratios for each period. Participants were censored at age 65 or at their last interview before age 65.”

**Results, page 20, paragraph 2:** “Over the follow-up period, 95 War Babies developed persistent CIND, with an incidence rate of 6.45 per 1,000 person-years. Among Early Baby Boomers, 115 cases of persistent CIND were observed (5.30 per 1,000 person-years), and among Mid Baby Boomers, there were 178 cases (4.86 per 1,000 person-years).

3. My primary concern is the application of the cognitive status algorithm to a relatively young cohort (ages 50–54). According to Petersen et al., the prevalence of mild cognitive impairment among individuals aged 60–64 is approximately 6.7%. Therefore, the finding that approximately 20% of participants aged 50–54 (1,935 out of 10,896) were classified as having CIND or dementia at baseline appears highly implausible and suggests potential misclassification. This raises substantial concerns about the validity of the algorithm when applied to younger age groups. The algorithm was originally developed and validated using data from ADAMS, which seems to have included only individuals aged 70 and older. I recommend the authors provide strong justification for the algorithm’s use in this age group and whether such a high number is realistic or consider alternative approaches more appropriate for middle-aged adults.
   Petersen RC, Lopez O, Armstrong MJ, Getchius TSD, Ganguli M, Gloss D, Gronseth GS, Marson D, Pringsheim T, Day GS, Sager M, Stevens J, Rae-Grant A. Practice guideline update summary: Mild cognitive impairment: Report of the Guideline Development, Dissemination, and Implementation Subcommittee of the American Academy of Neurology. Neurology. 2018 Jan 16;90(3):126-135. doi: 10.1212/WNL.0000000000004826. Epub 2017 Dec 27. PMID: 29282327; PMCID: PMC5772157.
   a. **Response:** We thank the reviewer for these helpful comments and now provide additional justification for this approach. First, we now include a flowchart which corrects the order of the study exclusions in the original submission. In the original submission, the numbers reflected exclusion of individuals by HRS cognitive test score before we excluded individuals by age at interview. However, we actually excluded individuals by age at interview before exclusion by HRS cognitive test score. After this correction, the number of individuals excluded due to an HRS cognitive test score <12 is 1,236, rather than 1,935. This represents a prevalence of 13.6% (unweighted) of individuals with a score <12 (1,236/9,088; Figure 1 shows the updated flowchart). After weighing, the prevalence is 10.1%. Of note, this does not represent the prevalence of individuals with “persistent CIND,” which we defined as a score <12 at two subsequent waves to increase the likelihood of correct classification. Instead, it represents a more heterogeneous group of individuals whose cognitive score was <12 at a single wave.

As the reviewer notes, this score threshold was originally validated for people ages 70 or older. However, prior research by Choi et al found that use of this score cut-off to classify CIND status results in increasing prevalence of CIND across 5-year age bands (i.e., 10.7% among those ages 55-59, 11.9% among those ages 60-64, 14.1% among those ages 65-69), suggesting good concurrent validity in younger age groups. Additionally, a recent nationally representative analysis of HRS data that defined mild cognitive impairment using the Harmonized Cognitive Assessment Protocol demonstrated that 22% of 65-69 year-olds had MCI, suggesting that a 10.1% prevalence among individuals ages 50-56 is plausible. Also of note, the Petersen article is a meta-analysis which does not nationally represent middle-aged individuals. The included studies also used varying definitions of MCI, and the range of reported estimates vary with differences in criteria, making comparisons across studies challenging. We have edited the manuscript to update the flowchart and to provide further justification, as follows:

**Results, page 7, paragraph 2:** “Our analytic sample included participants who were ages 50-56 on enrollment in the 1998, 2004, or 2010 HRS survey waves. We excluded participants enrolled in 1992 because different cognitive measures were used in the 1992 and 1994 waves of HRS. We also excluded participants enrolled in 2016 due to the limited follow-up waves currently available; while available data extend through 2020, we excluded 2020 data due to the impact of the COVID-19 pandemic on in-person data collection. For participants enrolled in 1998, 2004, and 2010, respectively, we used a consistent follow-up period (1998-2006, 2004-2012, and 2010-2018). Of 10,296 participants in these waves who were ages 50-56 at enrollment, we excluded 5 who were in a nursing home at baseline, 1,203 who had a zero-sampling weight, which is typically due to being a spouse younger than age 50 of an HRS participant, and 1,236 who had an HRS cognitive score consistent with CIND or dementia at enrollment. Our final analysis included longitudinal data from 7,852 participants, collected at approximately 2-year intervals through 2018 (Figure 1).”

**Results, page 8, paragraph 2:** “Of note, this score threshold was originally validated for persons ages 70 and older. However, prior research has found that use of this cut-off to classify CIND status results in increasing prevalence of CIND across 5-year age bands (i.e., 55-59, 60-64, 65-69), suggesting good concurrent validity in younger age groups.”

4. It is not clear whether the “key covariates” included in the analysis are intended to be treated as confounders, effect modifiers, or whether included for other reasons. Could some of these be mediators? Conceptually, variables included in the model to control for bias in the exposure-outcome relationship should be referred to as confounders. The generic label “covariate” lacks clarity. Moreover, presenting and interpreting effect estimates for covariates included to adjust for confounding is discouraged. Specifically, paragraph 4 in the Group-based trajectory models section and paragraph 2 (and Table 2) in the Linear-mixed effects models section display and interpret effect estimates for confounding variables. For guidance, please see: Westreich et al. for more details: Westreich D, Greenland S. The table 2 fallacy: presenting and interpreting confounder and modifier coefficients. Am J Epidemiol. 2013 Feb 15;177(4):292-8. doi: 10.1093/aje/kws412. Epub 2013 Jan 30. PMID: 23371353; PMCID: PMC3626058.
   a. **Response:** We agree that this is an important issue that needs further discussion. Building on the expanded introduction which discusses the putative role of lower levels of SES in recent health declines in middle-aged adults, we now include a conceptual model (Figure 2) to show the proposed causal pathway leading from SES to chronic health conditions and cognitive outcomes. Because the included variables are mediators in this proposed causal pathway, we have retained the effect estimates. We have updated the methods as follows:

**Methods, page 10, paragraph 2:** “*Main Exposure and Other Measures*

The primary exposure was birth cohort. We included three birth cohorts in our analysis, enrolled in 1998, 2004, and 2010, respectively: War Babies (born 1942-1947); Early Baby Boomers (born 1948-1953); and Mid Baby Boomers (born 1954-1959).

We also considered several key additional variables based on prior literature and guided by the Lancet Commission life course model of dementia risk and the MacArthur Network on Socioeconomic Status and Health conceptual framework (Figure 2) (2, 44-46). The Lancet Commission model was developed to inform population-level studies of dementia risk and provides a framework for examining how risk and protective factors from different phases of the life course affect cognition. Because lower SES appears to play a role in worsening health status in middle-aged individuals in more recent birth cohorts, our conceptual model also draws on the work of the MacArthur Network on Socioeconomic Status and Health, which has explored the role of SES in health disparities and how SES may influence health outcomes.(45, 46) SES reflects an individual’s economic and social position and is typically characterized by several measures, include education, income, and net worth. In their evidence-based model, the MacArthur Network proposes that the components of SES influence health outcomes via their effect on downstream mediators, including environmental exposures, health behaviors, access to health care, and psychosocial attributes and impacts, including stress. In turn, these factors lead to an increased risk of chronic conditions, which are known risk factors for CIND and dementia. The current study focuses on understanding how cognitive outcomes vary across birth cohorts, rather than on examining mechanisms of cognitive decline or identifying novel risk factors. For this reason, we focus on variables that are established risk factors for cognitive decline in this proposed causal pathway and have been extensively examined in prior studies examining trends in the prevalence of cognitive impairment among older adults, including sociodemographic characteristics, measures of SES, and measures of health status (2, 44). Self-reported sociodemographic characteristics at study entry included age, gender, and race/ethnicity, and measures of SES included educational attainment and household net worth, calculated based on self-reported assets minus debts and adjusted for inflation using the Consumer Price Index for all Urban Consumers (CPI-U) (47). Measures of health status included self-reported medical conditions (hypertension, stroke, diabetes, cardiac disease) and body mass index, calculated using self-reported weight and height.”

**Figure 2:** Please see revised manuscript.

5. In addition, the authors might consider excluding the p-values for differences in Table 1. Please see: Hayes-Larson E, Kezios KL, Mooney SJ, Lovasi G. Who is in this study, anyway? Guidelines for a useful Table 1. J Clin Epidemiol. 2019 Oct;114:125-132. doi: 10.1016/j.jclinepi.2019.06.011. Epub 2019 Jun 20. PMID: 31229583; PMCID: PMC6773463.
   a. **Response:** We now exclude the p values from Table 1, as suggested.
6. The Cox model needs to be specified in more detail. It is unclear what was used as the time axis - age or follow-up time? Was the model left-truncated?
   a. **Response:** We have now specified the Cox model in more detail. Specifically, we have clarified that the time axis is time since baseline, rather than age. In addition, we have clarified that the participants were right-censored, as follows:

**Methods, page 12, paragraph 2:** “We accounted for the competing risk of death in two ways. First, we censored at the time of death to model cause-specific hazard in Cox regressions; second, we used Fine and Gray’s subdistribution hazard model (48). For the Cox regression models, the time axis was time since baseline, and participants were right-censored by death or end of follow-up.”

7. Similarly, the linear mixed-effects model needs to be described in more detail. It is not specified whether time was modelled as age or as years since baseline. If time was modeled as years since baseline, it is unclear whether there was an overall cognitive decline observed in the cohort. Further, it appears that interaction terms between time and each covariate were included (in addition to interactions between birth cohort and selected covariates listed in methods). If so, this likely increases model complexity. A more parsimonious model may be preferable.
   a. **Response:** We have also specified the linear mixed-effects model in more detail. We now specify that time was modeled as years since baseline. The overall slope estimated from the mixed effects model was -0.083 (standard error, 0.006), indicating an overall decline in cognitive function. We have also clarified that the interaction terms between time and each covariate were included in the model. While this adds complexity, these terms were necessary to account for the effects of covariates on the slope of cognitive function over time. We have edited the methods as follows:

**Methods, page 12, paragraph 1:** “After confirming that the trajectories could be approximated by a straight line, we next fit linear mixed-effects models by assuming a random intercept and random slope for each participant with slope indicating the rate of cognitive decline. The intercepts and slopes were assumed to follow normal distributions. In linear mixed-effects models, time was modeled as years since baseline.”

8. What was the proportion of missing data in the covariates? And how were missing data handled? It would be helpful to have a flow diagram illustrating how the final analytic sample was derived from the whole HRS cohort.
   a. **Response:** We now provide the proportion of missing data in each of the covariates in the text, which ranged from 0% to 1.49%. We further clarify that because the proportions of missing data were low across the covariates, we used complete case analysis, in which we excluded those participants with missing data. We have also created a flow diagram to show how the final analytic sample was derived from the overall HRS cohort, as also discussed in Response #3 above. We have included these additions in the revised manuscript, as follows:

**Methods, page 13, paragraph 1:** “The proportion of missing data in the variables was 0% with the exception of race/ethnicity (0.22%), educational attainment (0.04%), and BMI (1.49%). Due to the low percentage of missingness, we used complete case analysis in which we excluded those participants with missing data.”

**Figure 2: (Drafted in PPT)** Please see revised manuscript.

9. Were any participants categorized as having dementia during follow-up? If so, how were they treated? What was the number of CIND events during the follow-up period?

**Response:** Thirty-five participants out of the 388 persistent CIND cases were classified as having dementia based on their TICS scores or proxy responses. However, in time-to-event analyses, we now clarify that we first identified the initial episode of either CIND *or* dementia based on self- or proxy-reported cognitive measures. We then imputed time to CIND based on the date when CIND or dementia was first reported. To increase the likelihood of correct classification, we defined persistent CIND as two or more consecutive interviews indicating CIND or dementia, with time to persistent CIND calculated from baseline to first occurrence. We assumed a linear cognitive function change between the dates of interview before and after CIND or dementia and interpolated the actual CIND or dementia occurrence date. We have now revised the methods to include the number of individuals who were categorized as having CIND or dementia over follow-up and to clarify how dementia was treated in analyses:

**Methods, page 10, paragraph 1:** “We also examined incidence of persistent CIND, assessed as a time-to-event outcome. To do so, we first identified the initial episode of CIND or dementia based on self- or proxy-reported cognitive measures. To increase the likelihood of correct classification, we defined persistent CIND as two or more consecutive interviews indicating CIND or dementia (43), with time to persistent CIND calculated from baseline to first occurrence. We assumed a linear cognitive function change between the dates of interview before and after CIND or dementia and interpolated the actual CIND occurrence date.”

**Results, page 20, paragraph 1:** “Over the follow-up period, 95 War Babies developed persistent CIND, with an incidence rate of 6.45 per 1,000 person-years. Among Early Baby Boomers, 115 developed persistent CIND (5.30 per 1,000 person-years), and among Mid Baby Boomers, 178 developed persistent CIND (4.86 per 1,000 person-years).”

10. I appreciate that the authors considered the competing risk of death in their analyses. The consistency of the results across these models adds confidence to the robustness of the findings. However, would it be possible to include more details on mortality patterns in the descriptive statistics?
    a. **Response:** We have now provided information on the number of deaths over follow-up in the descriptive statistics, as follows:

**Results, page 20, paragraph 2:** “Seventy-six War Babies died over follow-up, 114 Early Baby Boomers died, and 104 Mid Baby Boomers died.”

11. What was the mean (or median) length of follow-up.
    a. **Response:** We now provide the weighted median length of follow-up for each birth cohort in the results section, as follows:

**Results, page 20, paragraph 2:** “The weighted median follow-up was 7.9 years (IQR, 7.5, 8.2) among War Babies, 7.9 years (IQR, 7.6, 8.2) among Early Baby Boomers, and 7.4 years (IQR, 6.1, 8.0) for Mid Baby Boomers.”

12. Please include number of participants (N) in Tables 2 and 3 and the corresponding supplemental tables.
    a. **Response:** We now include the number of participants in the table header for Tables 2 and 3 and in the corresponding supplemental tables. Please see these updated Tables in the manuscript and supplement.
13. The title suggests a follow-up period of 20 years, which may be misleading. The maximum follow-up within any single birth cohort was 8 years. I understand that the 20-year figure refers to the total span of HRS data across all cohorts, but I would recommend revising the title to avoid potential confusion.
    a. **Response:** We agree and have revised the title accordingly. The title now reads, “Trends in Cognitive Outcomes in Middle-Aged Americans Across Three Birth Cohorts.”

We thank the Editors and Reviewers for their time and efforts to improve our manuscript.

Sincerely,

Rebecca Brown, MD, MPH

Associate Professor of Medicine, Division of Geriatric Medicine

Perelman School of Medicine of the University of Pennsylvania

1.	Choi H, Schoeni RF, Martin LG, Langa KM. Trends in the Prevalence and Disparity in Cognitive Limitations of Americans 55-69 Years Old. J Gerontol B Psychol Sci Soc Sci. 2018;73(suppl_1):S29-s37.
