# Supplementary material for: Trends in cognitive outcomes in middle-aged Americans across three birth cohorts
Source: PLoS One. 2025 Dec 5;20(12):e0338368. doi: 10.1371/journal.pone.0338368 (PMC12680256; doi:10.1371/journal.pone.0338368)
Supplement: Attachment — Submitted filename: ADRD MS Resubmission Response Letter 2.docx [file pone.0338368.s010.docx]

Rebecca T. Brown, MD, MPH

Associate Professor of Medicine

3615 Chestnut Street

Philadelphia, PA 19104

Tel: (215) 573-2021

Rebecca.Brown@pennmedicine.upenn.edu

September 3, 2025

Amruta Deshpande, Academic Editor
*PLOS One*

Dear Dr. Deshpande,

We thank the Editor and Reviewers for their comments and appreciate the opportunity to address the comments and revise our manuscript. Below, please find item-by-item responses to the comments, which are included verbatim. All page, paragraph, and line numbers refer to locations in the revised manuscript and online-only supplement. We have uploaded a clean copy of the revised manuscript as well as a copy of the manuscript with tracked changes.

**Journal requirements:**

1. Please include the following items when submitting your revised manuscript:

- A rebuttal letter that responds to each point raised by the academic editor and reviewer(s). You should upload this letter as a separate file labeled 'Response to Reviewers'.
- A marked-up copy of your manuscript that highlights changes made to the original version. You should upload this as a separate file labeled 'Revised Manuscript with Track Changes'.
- An unmarked version of your revised paper without tracked changes. You should upload this as a separate file labeled 'Manuscript'.

a. **Response:** We have included the requested documents in our resubmission.

**Responses to Reviewer 3:**

1. Dear Author,
   The research work certainly brings out, output with rigorous approach, however, to add more strength to the discussion work following points are suggested:
   1. Bringing academic knowledge discussion part on the conceptual model deeply by discussing each causal pathway relationship and its implication to medical and healthcare fraternity in macro view to both research field and medical fraternity policy makers and experts for their utility.
   a. **Response:** We thank the reviewer for this comment and opportunity to further discuss the conceptual model and its implications, and we have edited the discussion accordingly. Of note, the current study focuses on understanding how cognitive outcomes vary across birth cohorts, rather than on examining mechanisms of cognitive decline or identifying novel risk factors (see Methods, page 11, paragraph 1). For this reason, the study includes variables that are established risk factors for cognitive decline in the proposed conceptual model and have been included in prior studies of trends in cognitive outcomes over time. These include sociodemographic characteristics, measures of socioeconomic status, and measures of health status. For this reason, the discussion does not consider every pathway in the conceptual model, as not all of these are relevant to the current manuscript. However, we have added additional discussion of the relevant pathways in the conceptual model to the discussion, as follows:
   b. **Page 24, paragraph 1: “The conflicting findings of the current and prior study may reflect differences in birth cohort categorization: we examined 5-year birth cohorts, which distinguish between those born in the early versus middle Baby Boom (1948-1953 vs 1954-1959), while 10-year cohorts combine those individuals. We found key differences in baseline socioeconomic status and health status between 5-year cohorts, with Mid Boomers having lower educational attainment and net worth and a higher prevalence of obesity and several chronic medical conditions compared to those born in earlier cohorts. These findings are consistent with the pathways described in our conceptual model, which shows how upstream SES may “get under the skin” by influencing downstream exposures in the social and physical environment, ultimately leading to a higher prevalence of chronic health conditions that are important risk factors for AD/ADRD. These findings are also consistent with prior studies showing that those born in the mid to later part of the Baby Boom experience worse health outcomes than those born earlier in the Baby Boom, possibly due to an “Easterlin cohort effect” (55-57) Similar to the conceptual model, this effect posits that due to the large generation size of Baby Boomers, those born later faced increased competition in the job market and lower socioeconomic status at adulthood, in turn leading to increased stress and poorer health outcomes across the life course. The finding that more recent birth cohorts had slower rates of cognitive decline despite a higher prevalence of chronic conditions suggests that their higher levels of education may have buffered the rate of decline from a lower cognitive baseline, possibly combined with increased availability of medical treatments and technologies that can address conditions such as diabetes, hypertension, and cardiac disease** (45, 58).”
2. If we can propose benefits of this model to the stakeholders of medical and healthcare field.
   a. **Response:** We agree that further discussion of the benefits of the model for the medical and health care fields would strengthen the discussion. We have highlighted key benefits of using a conceptually informed approach, as follows:
   b. **Page 26, paragraph 1:** **“Taken together, our findings suggest how the interplay of risk factors across birth cohorts may contribute to differences in cognitive outcomes. Our findings and those of prior studies support the key importance of early life education as well as cognitive stimulation across the life course in developing “cognitive reserve” that buffers against mid- and later life cognitive decline (2). In the current study, lower educational attainment was the strongest risk factor for cognitive decline across analyses of both cognitive trajectories and incidence of CIND. Race/ethnicity was also strongly associated with cognitive outcomes; this association likely reflects the influence of factors not accounted for in our models, which may include educational quality and exposure to childhood adversity and aspects of the physical and social environment. Our conceptual model shows the pathways, from SES, to environmental exposures, to chronic health conditions, through which such exposures may influence downstream cognitive outcomes. Of note, although individuals identifying as Black non-Latino, Latino, and of another race/ethnicity had lower baseline cognitive scores in mixed effects models compared to individuals identifying as White, rates of cognitive decline were similar across racial/ethnic groups, suggesting that disparities in cognitive outcomes may have their roots in earlier life experiences and exposures, consistent with the findings of prior work (59, 60). These findings suggest the importance of ongoing research to elucidate the complex pathways through which SES may influence cognitive outcomes in middle age via exposures in the social and physical environment. Understanding these pathways can help support policy efforts to bolster educational attainment and also inform the development of interventions to reduce the impact of low SES on health outcomes. These findings also underline the importance for policy makers of supporting and promoting interventions to increase educational attainment and educational quality.”**

We thank the Editors and Reviewers for their time and efforts to improve our manuscript.

Sincerely,

Rebecca Brown, MD, MPH

Associate Professor of Medicine, Division of Geriatric Medicine

Perelman School of Medicine of the University of Pennsylvania
